# Letter to the Editor: Catheter-Based Renal Denervation to Treat Chronic Kidney Pain Secondary To Obstructive Renal Disease

**DOI:** 10.1007/s00270-025-04132-5

**Published:** 2025-07-25

**Authors:** Haseeb Mukhtar, Sanjay Misra

**Affiliations:** Department of Radiology, Mayo Clinic, 200 First Street SW, Rochester, MN 55905 USA

Catheter-based renal denervation (RDN) has been successfully used for resistant hypertension [1], and more recently, studies have documented its utility in managing kidney-related pain [2, 3]. We present a case of chronic renal pain secondary to obstructive renal disease managed with catheter-based RDN.

A 28-year-old female presented with chronic bilateral flank pain, more pronounced on the right. The pain, rated 4–5/10, was dull in nature with intermittent sharp episodes, radiated to the anterior abdomen, worsened by urinary tract infections (UTIs), and partially controlled with acetaminophen and oxycodone.

Her medical history included primary hyperparathyroidism secondary to an ectopic parathyroid adenoma, recently resected. Complex urological history included: recurrent renal calculi, hydronephrosis, bilateral vesicoureteral reflux, recurrent UTIs, pyelonephritis, renal abscess, and bilateral infundibular stenosis and progressive renal dysfunction with chronic kidney disease stage 3b. Past procedures include deflux injections, ureteroscopy, percutaneous nephrolithotomy, and infundibular laser treatment. She was an active smoker.

Recent estimated glomerular filtration rates were 38 mL/min/1.73 m^2^ (creatine based) and 27 mL/min/1.73 m^2^ (cystatin C based). Serum creatinine was 1.85 mg/dL, and blood urea nitrogen was 17 mg/dL. Post-parathyroidectomy, the serum calcium and parathyroid hormone levels normalized.

Despite prior urological interventions, her pain persisted. She was referred for catheter-based RDN after informed consent. Access was gained into the right common femoral artery under ultrasound guidance. A 6F catheter was introduced, and renal angiograms showed single renal arteries bilaterally. An SOS catheter was used to select the left renal artery and was replaced by a 7F Ansel I sheath. A Recor Paradise® catheter of 4.2 mm balloon size was used to treat the artery in two separate locations. Subsequently, the right renal artery was selected and treated with a 3.5-mm balloon size Paradise® catheter at two different locations again. Post-treatment angiograms showed preserved renal perfusion.

At 1-month follow-up, the right flank pain was gone; there was some left flank pain that was tolerable, no longer requiring pain medications. The next follow-up at 5 months did not reveal any significant change in the appearance of the dilated renal calyces and intrarenal calculi on ultrasound, but the flank pains had resolved. The eGFR calculated using creatinine and cystatin C had increased to 44 mL/min/1.73 m^2^ and 33 mL/min/1.73 m^2^, respectively. The serum creatinine had come down to 38 mg/dL, and BUN was 15 mg/dL at this follow-up. The renal function was stable at 1-year.

This case report utilized catheter-based RDN to treat pain secondary to chronic obstructive renal disease and demonstrated efficacy at the follow-up visits. Obstructive renal disease is an important cause of chronic renal pain [2]. While the primary focus is treatment of the underlying cause (nephrolithiasis, ureteric strictures, etc.), this is not always successful as shown in our case. Past literature has also supported the efficacy of catheter-based renal denervation using radiofrequency catheters systems, namely Symplicity® (Medtronics) and Vassix® (Boston Scientific), respectively [3, 4]. While a single case comes with its limitations, this letter signifies that ultrasound-based RDN systems can be used to treat obstructive disease-related chronic kidney pain that is refractory to primary treatment measures. It also highlights the safety of this method as demonstrated by the improvement in renal functions post-operatively.
